# Identification of Selective ATP-Competitive CMG Helicase Inhibitors for Cancer Intervention that Disrupt CMG-Replisome Function

**DOI:** 10.21203/rs.3.rs-3182731/v1

**Published:** 2023-08-11

**Authors:** Shengyan Xiang, Xingju Luo, Darcy Welch, Damon R. Reed, Mark G. Alexandrow

**Affiliations:** 1 Cancer Biology and Evolution Program, Moffitt Cancer Center and Research Institute, Tampa, FL 33612; 2 Molecular Oncology Department, Moffitt Cancer Center and Research Institute, Tampa, FL 33612; 3 Department of Individualized Cancer Management, Moffitt Cancer Center and Research Institute, Tampa, FL 33612

**Keywords:** CMG helicase, MCM, Cdc45, GINS, DNA replication, aminocoumarin

## Abstract

The human CMG helicase (Cdc45-MCM-GINS) is a novel target for anti-cancer therapy due to tumor-specific weaknesses in CMG function induced by oncogenic changes and the need for CMG function during recovery from replicative stresses such as chemotherapy. Here, we developed an orthogonal biochemical screening approach and identified selective CMG inhibitors (CMGi) that inhibit ATPase and helicase activities in an ATP-competitive manner at low micromolar concentrations. Structure-activity information and *in silico* docking indicate that CMGi occupy ATP binding sites and channels within MCM subunits leading to the ATP clefts, which are likely used for ATP/ADP ingress or egress. CMGi inhibit cell growth and DNA replication using multiple molecular mechanisms. CMGi block helicase assembly steps that require ATP binding/hydrolysis by the MCM complex, specifically MCM ring assembly on DNA and GINS recruitment to DNA-loaded MCM hexamers. During S-phase, inhibition of MCM ATP binding/hydrolysis by CMGi causes a ‘reverse allosteric’ dissociation of Cdc45/GINS from the CMG that destabilizes the replisome and disrupts interactions with Ctf4, Mcm10, and DNA polymerase-α, -δ, -ε, resulting in DNA damage. These novel CMGi are selectively toxic toward tumor cells and define a new class of CMG helicase-targeted anti-cancer compounds with distinct mechanisms of action.

## Introduction

The replicative CMG helicase is an emerging target for anti-cancer intervention due to exploitable vulnerabilities in cancer cells resulting from oncogene-driven mismanagement of CMG assembly and function ^1^. However, to date, no small chemical inhibitors of the human CMG helicase have been identified. The CMG is a multi-subunit enzyme that performs the primary DNA melting and unwinding steps within replisomes during DNA replication in eukaryotic cells ^2^. The CMG helicase is composed of Cdc45, a Mini-Chromosome Maintenance (MCM) heterohexameric ATPase core, and the GINS tetramer (*Go-Ichi-Ni-San* in Japanese for Sld5, Psf1, Psf2, and Psf3)^2, 3, 4^. Assembly and activation of the CMG occur in a stepwise manner, with an excess of MCM hexamers loaded onto DNA during G1 phase (called Licensing ^5, 6^), followed by recruitment of Cdc45/GINS near the G1-S transition to a subset of these MCM hexamers ^3, 4, 7, 8^. The dynamics of this MCM-CMG conversion process are important for maintaining genome stability in human cells ^1^. Unused MCM complexes act as reserves that are converted to CMG helicases during replicative stress to facilitate recovery of DNA replication ^1, 9, 10, 11, 12^. Reserve MCM complexes also modulate replication fork speeds to prevent DNA damage ^13^.

Oncogenic changes cause problems with CMG assembly and function ^1^. Cyclin E overexpression reduces the number of MCM hexamers that load onto DNA, resulting in reduced MCM reserves, loss of replication fork fidelity, and consequent DNA damage ^1, 14^. Elevated Myc over-stimulates the conversion of MCM hexamers into CMG helicases, leading to DNA damage in genomic regions with excessive CMG activity due to increased replication fork density and reduction of unused MCM reserves ^1, 15, 16, 17^. While these oncogenic events produce DNA damage that facilitates tumorigenesis and tumor heterogeneity, they also create a reduction in MCM/CMG functional fidelity in tumor cells that is likely exploitable with CMG inhibitors ^1^. In addition, inhibition of MCM/CMG reserves can selectively sensitize tumor cells to fork-stalling chemotherapy drugs ^11, 12^, suggesting that CMG inhibitors will have the potential to overcome chemo-resistance in the management of cancer.

We report here the design of an orthogonal chemical screening approach and its use in the identification of selective ATP-competitive inhibitors of the ATPase and helicase activities of the human CMG enzyme (CMGi). These CMGi have drug-like features and are members of the aminocoumarin class of compounds ^18^, specifically clorobiocin and coumermycin-A1. These CMGi inhibit the CMG by occupying multiple Mcm2-7 ATP-binding clefts and channels, the latter of which are likely used by ATP to access ATPase domains within the MCM ring. CMGi display distinct modes of action for cell growth inhibition and induction of DNA damage relative to other chemotherapy drugs, blocking MCM DNA binding and GINS recruitment during CMG assembly, and disrupting CMG and replisome co-structural integrity during S-phase. Tumor cells are selectively sensitive to these CMGi, supporting that the CMG helicase is a tumor-specific vulnerability and novel anti-cancer target. These CMGi provide unique probes to investigate ATPase-dependent CMG/MCM functions in human cells and can inform the development of a new class of anti-cancer compounds that target and disrupt CMGs/replisomes.

## Results

### Identification of Human CMG Helicase Inhibitors (CMGi)

**Figure 1:**
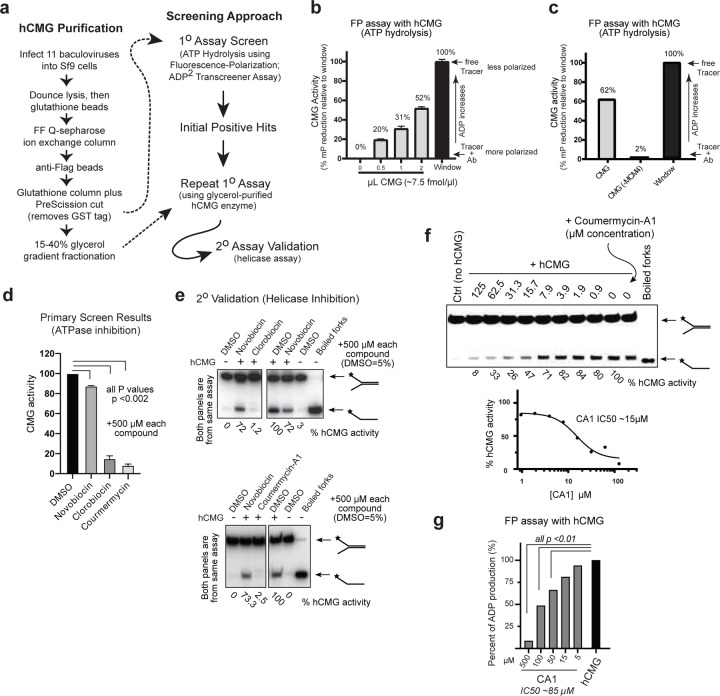
Identification of Human CMG Helicase Small Chemical Inhibitors (CMGi). (**a**) Purification of active human CMG helicase (hCMG) and steps involved in screening for hCMG inhibitors using orthogonal biochemical assays. (**b**) hCMG helicase assessed in primary ATPase assay to determine amounts of hCMG necessary for chemical library screening (10 *µ*L reactions). The fluorescent-polarization (FP) window is determined using analytes from the screening assay without added enzyme. (**c**) FP ATPase assays measuring hCMG activity from a preparation of hCMG holo-helicase compared to a parallel preparation of hCMG lacking co-expression of Mcm4. The assay compared ~15 fmol/2 *µ*L hCMG to the same amount of sample from hCMG(-Mcm4). (**d**) Small chemical inhibitors of hCMG ATPase activity were identified in primary screening at 1 mM chemical concentrations, repeated at 500 *µ*M. The DMSO solvent was compared as a control. (**e**) Potential hCMG inhibitors identified in the primary assay assessed in a secondary fork-unwinding assay measuring effects on hCMG helicase activity. Clorobiocin (top panels) and coumermycin-A1 (CA1; lower panels) were compared to novobiocin. (**f**) Fork-unwinding assay with purified hCMG helicase determined the IC50 for helicase inhibition by CA1. (**g**) FP ATPase assay measuring hCMG activity in presence of increasing [CA1]. Percent change was based on a comparison to an ADP-ATP standard curve.

We developed a rigorous biochemical screening approach utilizing two rounds of primary screening with a commercially available ATP hydrolysis assay and a secondary orthogonal validation assay that measures DNA unwinding by the human CMG (hCMG; Figure 1A). The hCMG helicase was purified using the established protocol of Hurwitz and colleagues in which all 11 hCMG subunits are co-expressed using baculoviral-based infections of insect cells followed by a multistep purification of the hCMG holoenzyme (Figure 1A; detailed approach described in Online Methods)^19, 20^. The quality of the hCMG enzyme obtained was verified by silver staining and immunoblotting, which showed that all 11 hCMG subunits were present at similar stoichiometries and purity compared to that obtained in previous studies (Supplemental Figure S1A&B)^19, 20^. The hCMG isolated is active in DNA fork-unwinding (helicase) assays and is dependent on the binding and hydrolysis of ATP as indicated by a dose-dependent suppression of fork-unwinding activity in the presence of slow-hydrolyzable ATP-γ-S (Supplemental Figure S1C). For fork-unwinding activity, the hCMG displays an ~K_m_ of 690 *µ*M [ATP] (see below), in close agreement with the K_m_ (625 *µ*M [ATP]) for hCMG ATP hydrolysis activity described by others ^19, 20^. The specific ATPase activity of the isolated hCMG also closely matches that obtained by Hurwitz and colleagues (see Methods)^19, 20^.

We optimized the ADP^2^ Fluorescent-Polarization (FP) Transcreener Assay (BellBrook Labs, Madison, WI) for quantitative analysis of ADP production by the hCMG (see Methods and Supplemental Figure S2A–C). The assay relies on a patented anti-ADP^2^ antibody that binds and polarizes an ADP-Tracer, changes to which due to competition with ADP produced by the hCMG are measurable in FP plate readers. This assay is highly sensitive and reliable (Z’ >0.6; Supplemental Figure S2B), being able to quantify small changes in ADP production^21, 22, 23, 24^. We titrated purified hCMG into the assay, which produced a dose-dependent increase in ADP production (Figure 1B). The hCMG concentration that produced an ~50% change in the assay window was used for screening. Because significant quantities of hCMG are required for screening, we performed primary chemical library screening with hCMG purified through the Flag enrichment step, and a repeat of primary screening with positive hits on a higher purity (but lower yield) hCMG after glycerol fractionation (Figure 1A). The hCMG obtained after the Flag enrichment step is active in the primary assay and is dependent on the presence of intact hCMG helicase, as failure to express Mcm4 yields preparations devoid of ATPase activity (Figure 1C). The latter indicates that a contaminating ATPase from insect cells is not present in our hCMG preparations during our screening.

We used the National Institutes of Health (NIH) Diversity Set VI library at 1 mM concentrations for primary screening, and repeated screening of positive hits at 500 *µ*M. Fewer than 3% of compounds were capable of partial or complete hCMG inhibition. One compound, clorobiocin, drew attention due to its drug-like features and effective nature of hCMG inhibition (Figure 1D). Clorobiocin did not interfere with far-red UV light readings or assay reagents. Clorobiocin is an aminocoumarin derived from *Streptomyces roseochromogenes* and is related to two similar chemicals, novobiocin and coumermycin-A1 ^18^. It was difficult to continue working with clorobiocin, as we needed fresh dry chemical powder for further validation work, the NIH had none available, and we could not find a commercial source. However, coumermycin-A1 (CA1) and novobiocin were commercially available, and we tested both for their ability to inhibit the hCMG. CA1 was found to be a potent inhibitor of ATP hydrolysis by the hCMG, while novobiocin had very little inhibitory effect on the hCMG (Figure 1D).

Validation with our secondary strand-displacement (helicase) assay showed that clorobiocin and CA1, but not novobiocin, were effective hCMG helicase inhibitors at 500 *µ*M concentrations (Figure 1E). The *in vitro* IC_50_ of CA1 for hCMG helicase inhibition was determined to be ~15 *µ*M (Figure 1F), which closely matches the IC_50_ of CA1 for reducing viability of human cells (see below). Using the FP assay and an ADP/ATP standard curve comparison, the IC_50_ for hCMG ATP hydrolysis inhibition is ~85 *µ*M (Figure 1G). The hCMG has six distinct ATPase clefts and it is likely that CA1 does not target all of them with the same efficiency (see below). It is therefore possible CA1 targets a cleft(s) necessary for helicase activity at higher affinity, but more CA1 is necessary to inhibit remaining ATP sites. Using yeast MCM complexes as the basis for screening, it has been suggested that ciprofloxacin might be an inhibitor of the human replicative helicase ^25, 26^. However, high concentrations of ciprofloxacin or other quinolones do not inhibit the purified hCMG helicase (Supplemental Figure S3A&B). We conclude that clorobiocin and CA1 represent the first biochemically-validated small chemical compounds that effectively inhibit ATPase and helicase activities of the hCMG (defined as CMGi).

### Coumermycin-A1 is an ATP-Competitive Inhibitor of hCMG Activity

**Figure 2:**
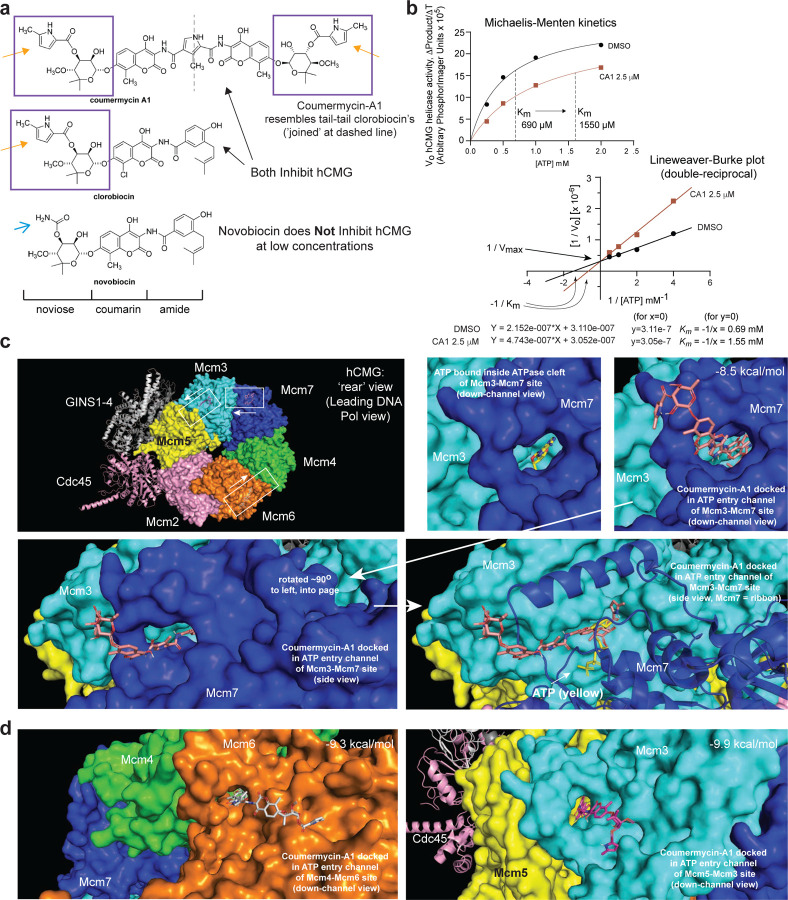
CMGi Inhibit CMG Helicase Activity Using an ATP-Competitive Mechanism. (**a**) Structures of coumermycin-A1 (CA1), clorobiocin, and novobiocin. Orange arrows denote 5-methylpyrrole group in the noviose sugar (purple boxes) of CA1 and clorobiocin, substituted with a carbamate in novobiocin (blue open arrow). (**b**) The kinetics of hCMG helicase activity were determined +/− CA1 (2.5 *µ*M) in increasing ATP concentrations, with results shown using Michaelis-Menten and double-reciprocal plots. Amount of ssDNA separated from radio-labeled DNA forks per 30 minutes was quantified. CA1 competes with ATP to inhibit hCMG helicase activity, raising the Km for [ATP]. (**c**) Structural image (top left) of the hCMG from publicly available cryo-EM (PDB accession code 6XTX). Inset boxes indicate regions enlarged in panels showing CA1 docked in channels leading to the ATPase clefts. The white arrow on each box denotes the direction of CA1 insertion. Top right (square panels), ATP bound in Mcm3-Mcm7 ATPase cleft and CA1 docked in the channel leading to Mcm3-Mcm7 ATPase cleft. Bottom left, side view of CA1 docked in Mcm3-Mcm7 channel. Bottom right, CA1 in Mcm3-Mcm7 channel partially overlapping position where ATP binds. (**d**) Left, CA1 docked in the channel leading to Mcm4-Mcm6 ATPase cleft. Right, CA1 docked in the channel leading to Mcm5-Mcm3 ATPase cleft. Docking was performed using Autodock and Pymol software, with predicted binding energies for CA1 calculated by software shown on each panel.

We next determined the mechanism of hCMG inhibition by CA1. The three aminocoumarins are comprised structurally of a noviose sugar head group joined to a coumarin group, and an amide group in two of the molecules (Figure 2A; amide domain referred to here as the ‘tail’ of the molecule)^18^. Clorobiocin and CA1 contain 2-methylpyrrole ester modifications to the sugar (Figure 2A, orange arrows) whereas novobiocin has a primary carbamate modification (Figure 2A, blue open arrow). CA1 resembles a tail-tail dimer of clorobiocin, but replaces the chlorine atom with a methyl group. Since novobiocin has little inhibitory effect on the hCMG in the biochemical assays (only at high concentrations), and largely differs from the other compounds in the structure of its sugar, this structure-activity relationship (SAR) indicates that the sugar head groups of clorobiocin and CA1 (Figure 2A, purple boxes) provide specificity in mediating inhibition of the hCMG.

Biochemical and co-crystallographic data assessing how these aminocoumarins inhibit gyrase (a type-II topoisomerase) provide information on how CA1 can inhibit the hCMG ^18, 27^. Aminocoumarins inhibit ATP binding and hydrolysis of gyrase using a competitive mechanism, inserting the sugar head groups through a channel/groove into the ATPase cleft of the GyrB subunit, with the sugar situated in the region where the adenosine and ribose of ATP normally interact ^18, 27^. CA1 interacts with two GyrB ATPase domains at the same time using this mechanism ^18, 27^. We reasoned that CA1 might likewise inhibit hCMG ATPase and helicase activities by direct competition with ATP binding and hydrolysis. We performed hCMG helicase assays to determine the mode of hCMG inhibition by CA1 in increasing ATP concentrations (Figure 2B). Michaelis-Menten kinetics and Lineweaver-Burke (double-reciprocal plot) analyses showed that CA1 inhibits hCMG helicase activity using a classic ATP-competitive mechanism, resulting in a significant increase in the K_m_ for [ATP] (690*µ*M without inhibitor to 1550*µ*M with CA1), without a change in the V_max_ of the hCMG.

We next used *in silico* docking of CA1 and clorobiocin as a means to model how these compounds interact with the ATPase domains of the hCMG to competitively block ATP binding and hydrolysis. The hCMG has six biochemically distinct ATPase domains formed between adjacent MCM subunit pairs ^4, 28, 29, 30^, and the cryo-EM structure of the hCMG has been determined^31^. Docking software places CA1 and clorobiocin into channels leading to the ATPase clefts of three MCM ATPase domains with similar binding energies, notably clefts for Mcm3-Mcm7, Mcm4-Mcm6, and Mcm5-Mcm3 (Figure 2C&D, CA1 docking; Supplemental Figure S4A–D, clorobiocin docking). Consistent with co-crystallographic data for aminocoumarin-gyrase interactions ^18, 27, 32, 33^, the sugar head groups are inserted into the ATP binding sites where adenosine and ribose from ATP are normally situated (‘sugar-first’ direction), while the coumarin group occupies channels leading to the ATP binding sites. CA1 can be docked in either direction in these MCM ATPase clefts/channels due to its symmetry. It is quite possible that these channels are used by ATP or ADP for ingress and/or egress during enzyme function, suggesting that clorobiocin and CA1 act like a ‘cork in a wine bottle’ to block ATP movement through the channels, consistent with their ATP-competitive mode of hCMG inhibition.

Three MCM ATPase domains were not capable of *in silico* docking for either compound, specifically the clefts for Mcm7-Mcm4, Mcm6-Mcm2, and Mcm2-Mcm5. In the existing hCMG structure^31^ the channels leading to these ATPase clefts are narrow relative to the MCM sites that can be docked, suggesting a steric hindrance to inhibitor binding (Supplemental Figure S4E&F). The cryo-EM structure of the hCMG is in one enzymatic state, and it remains possible that these sites might also be subject to inhibition by clorobiocin or CA1 under different enzymatic states when these channels might be accessible.

Novobiocin is similar in structure to clorobiocin and can also dock in the accessible MCM ATPase clefts, and in some cases the software places the novobiocin and clorobiocin in tail-first (sugar-out). However, these tail-first orientations are generally less favorable thermodynamically, and are not consistent with crystallographic information for gyrase binding (sugar-first), nor with CA1 binding being reliant on one of two identical sugar groups entering first ^18, 27, 32, 33^. Based on the sugar-first binding mode for clorobiocin and novobiocin, the docking software can estimate a binding affinity (interaction energy) and ~Kd for both related compounds. Using the Mcm3-Mcm7 channel/cleft as a model, the estimates for the two strongest-binding molecular poses for clorobiocin were −9.9 to −10.0 kcal/mol, and for novobiocin were −8.7 to −9.0 kcal/mol. This suggests an ~Kd of 89–105 nM for clorobiocin and 452–735 nM for novobiocin. Although these computational predictions must be interpreted with caution, the 5–7 fold lower Kd for clorobiocin is in agreement with biochemical assays that clorobiocin is a more effective inhibitor of the hCMG than novobiocin. Such specificity likely derives from the noviose sugar group specific to clorobiocin and CA1. However, overall hCMG inhibition by these compounds cannot be determined from analysis of a single cleft, but instead derive from a sum of inhibitor effects on multiple clefts that individually may or may not be required for specific hCMG/hMCM functions.

At present we do not know if certain MCM ATPase clefts display preferences for inhibitor binding over other clefts, particularly in cells, as determining this is technologically challenging for an enzyme comprised of six ATPase domains that are not easily separated for analysis. Going forward, we assess hCMG ATPase and helicase inhibition in cells with CA1 from a holo-enzyme perspective (ATP-competitive effects on all clefts combined) rather than a particular ATPase cleft.

### CMGi Inhibit MCM/Cdt1 Assembly on Chromatin

**Figure 3:**
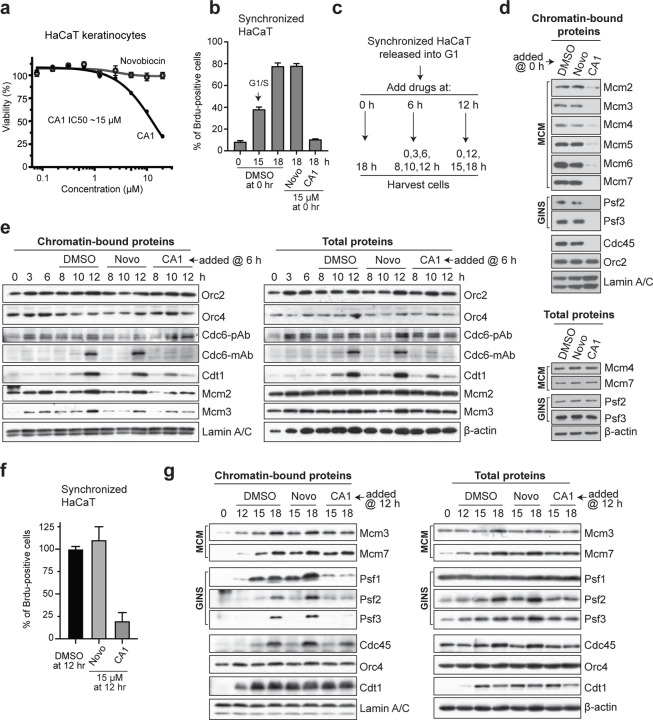
CMGi Inhibit Mcm2-7 ATPase-Dependent MCM Loading and CMG Assembly. (**a**) Cell viability (Titer-Glo) assays using HaCaT cells. Based on IC50 determination here for CA1, 15 *µ*M of CA1 or novobiocin was used in the next experiments. (**b**) HaCaT cells synchronized and released into G1 (time 0 hr) were assessed for DNA replication after exposure to compounds at time of release. BrdU-labeled cells were assessed at times indicated using immunofluorescence (IF) methods and three fields were averaged per condition, +/−1s.d. Note for HaCaT experiments in remainder of figures that 15 hrs is G1/S, and 18 hrs is early S-phase. (**c**) Experimental design for panels **d-g**. (**d**) Immunoblots of chromatin-bound or total proteins from synchronized HaCaT cells treated with compounds at time of G1 release (0 hr), and assessed at 18 hrs. (**e**) Immunoblots of chromatin-bound or total proteins from synchronized HaCaT cells treated with compounds at 6 hr (middle G1), and assessed at times indicated. (**f**) Synchronized HaCaT cells treated with compounds or DMSO in late G1 (12 hrs) and assessed for DNA replication using BrdU labeling at 18 hrs. Three fields of cells were averaged per condition, +/−1s.d. (**g**) Immunoblots of chromatin-bound or total proteins from synchronized HaCaT cells treated with compounds at 12 hr (late G1), and assessed at times indicated.

We used the immortalized, non-tumor derived human keratinocyte HaCaT cell line to assess effects of CMGi on growth, DNA replication, and CMG helicase assembly and function. HaCaT cells are synchronized in a quiescent state by serum deprivation and released into the cell cycle to study G1 and S-phase events. A cell viability analysis found that, while novobiocin has little effect, CA1 reduces viability with an IC_50_ of ~15 *µ*M (Figure 3A). Since this concentration of CA1 aligns with that required to inhibit *in vitro* hCMG helicase activity, and novobiocin has little effect, these results support that the hCMG is a major target of CA1 in human cells at these low concentrations. CA1 inhibits DNA replication when added to HaCaT cells in early G1, while novobiocin has little effect (Figure 3B), consistent with assembly and activity of hCMG complexes being required for progression into S-phase.

Studies of MCM assembly using yeast *in vitro* models have suggested that ATP binding and hydrolysis by most MCM ATPase clefts are required for efficient Mcm2-7 ring loading onto DNA ^29, 30^. We asked whether CMGi/CA1 could block chromatin/DNA binding of human MCM complexes *in vivo* due to a dependency on ATP utilization. HaCaT cells were synchronized and treated with CA1, novobiocin, or DMSO carrier in early-G1 (at release, 0 hrs), middle-G1 (6 hrs), or late-G1 (12 hrs) to assess effects of CMGi on different stages of MCM assembly (Figure 3C). Note the G1/S transition in HaCaT occurs at ~15 hrs after release (Figure 3B). MCM loading onto chromatin in human cells is significantly inhibited by early-G1 CA1 treatment (Figure 3D). GINS and Cdc45 loading onto chromatin is consequently blocked, while Orc2 chromatin binding is not affected. This suggests that the ORC complex, which contains ATPase domains required for its DNA-binding and roles in MCM loading, is not itself a target of CA1.

Some MCM complexes are already loaded onto chromatin between early and middle G1 (3–10 hrs after release), but an increase in MCM loading occurs around 12 hrs as cells approach G1/S (Figure 3E). While exposure of cells to CA1 at 6 hrs does not affect MCMs already loaded, the increase in MCM loading at 12 hrs is inhibited by CA1 but not by novobiocin. Cdt1 also loads onto chromatin at higher levels when MCM loading increases. However, CA1 blocks this Cdt1 loading and promotes loss of total Cdt1 (Figure 3E). Taken together with the previous experiment, these results indicate that CMGi inhibit an early step in the MCM loading process in human cells that requires efficient ATP binding and/or hydrolysis by Mcm2-7, but once loaded, MCMs are resistant to CMGi/CA1. The results also suggest that Cdt1 is sensitive to Mcm2-7 ATPase inhibition in human cells, consistent with yeast studies showing that MCM-Cdt1 interactions are adversely affected by defective Mcm2-7 ATPase sites ^29, 30^.

Orc2 and Orc4 affinity for chromatin is not affected by CA1 exposure in middle G1 (Figure 3E), again suggesting that the ATPases of ORC are not a target of CA1. Cdc6 protein is not affected in chromatin association by CA1 when measured using a polyclonal antibody (Figure 3E). In contrast, analysis with a monoclonal antibody to Cdc6 suggests that one form of Cdc6 increases on chromatin in parallel with MCM elevation and is sensitive to CA1. Cdc6 contains an ATPase domain that is not required for MCM assembly on DNA, but instead for removal of improperly loaded MCMs ^29, 30^. While we cannot rule out the possibility that the ATPase site of Cdc6 may be affected by CA1, these prior studies suggest that it is unlikely that this would contribute to the inhibition of MCM assembly we have observed in the presence of CA1.

### GINS Recruitment to DNA-loaded MCM Complexes is Inhibited by CMGi

HaCaT cells treated with CA1 in late-G1 (12 hrs), but not novobiocin, fail to undergo DNA replication (Figure 3F). Near the G1/S transition (15–18 hrs) there is additional MCM loading onto chromatin, which coincides with GINS and Cdc45 being recruited to loaded MCM hexamers on chromatin (Figure 3G). Treatment with CA1 during this late-G1 period has only a small effect, if any, on the remainder of MCM loading and does not affect Cdt1 dynamics, suggesting that Mcm2-7 ATPase functions in MCM/Cdt1 loading are no longer required at this later time. However, while Cdc45 is not appreciably affected, CA1 inhibits GINS recruitment to MCM hexamers (Figure 3G). Such results are consistent with yeast *in vitro* studies showing that certain ATPase sites of the Mcm2-7 ring are required for GINS binding^30^. However, our results differ somewhat from another yeast study showing that GINS and Cdc45 are both dependent on ATP binding to the Mcm2-7 ring^34^. Reasons for this difference may be that CA1 is less efficient at binding a particular MCM ATPase cleft involved in Cdc45 recruitment in human cells, or that the Cdc45 extraction conditions vary between experimental approaches. We conclude from these results that once MCMs have loaded, GINS recruitment in human cells requires ATP binding and/or hydrolysis by one or more ATPase sites of the Mcm2-7 ring and is inhibited by CMGi exposure.

### CMGi Are Selective for Mcm2-7 ATPases in Human Cells

**Figure 4:**
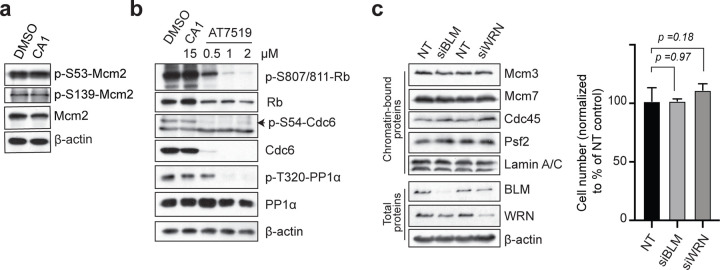
CMGi Are Selective hCMG Inhibitors in Human Cells. (**a,b**) Immunoblot of total proteins from synchronized HaCaT cells treated with compounds at time of release (0 hr) and collected after 18 hrs of exposure. DDK-dependent phosphorylation sites on Mcm2 were analyzed with phospho-specific antibodies in panel A. Cdk1- and Cdk2-dependent targets (Rb, Cdc6, and PP1α) were analyzed with phospho-specific antibodies in panel B. (**c**) Immunoblot of chromatin-bound or total proteins from asynchronous HaCaT cells treated 72 hrs with siRNA against Bloom or Werner helicases, or non-targeting control siRNA (NT). Graph on right is the quantification of cell numbers as a percent of NT-control after siBLM or siWRN exposure, +/−1s.d.

There are other enzymes with ATPase domains that function in MCM/CMG assembly, including Dbf4-Cdc7 (DDK) and Cdk2 ^2^. We asked whether CA1 affected these and other enzymes in human cells. DDK phosphorylates two sites in Mcm2 (S53 and S139) to facilitate Cdc45 recruitment ^35^, both of which show no phosphorylation changes after extended exposure to CA1 (Figure 4A). This agrees with our observation that Cdc45 is recruited to DNA-loaded MCM hexamers (Figure 3G) and indicates that DDK is not a target of CA1. A pan-Cdk inhibitor (AT7519) that efficiently targets Cdk1 (Cdc2), Cdk2, Cdk3, Cdk4, Cdk6, and Cdk9 blocks phosphorylation of Cdk2 targets, including Rb and Cdc6 (S54P) ^36 37^, and the Cdk1 target PP1α 38. However, extended exposure to CA1 has no effect on these substrates (Figure 4B). We conclude that CA1 does not target these kinases required for MCM/CMG assembly.

We next asked if two helicases (BLM and WRN) involved in DNA repair and recombination contribute to MCM/CMG assembly ^39^. Reduction of BLM or WRN using siRNA has no effect on MCM, Cdc45, or GINS recruitment to chromatin (Figure 4C, left), and has no effect on cell growth, indicating that they are not required for unperturbed DNA replication in HaCaT cells (Figure 4C, right). It is not known if CA1 can inhibit BLM or WRN. Since these data demonstrate that neither helicase is required for MCM/CMG assembly, any effects of CA1 on these helicases does not contribute to the outcomes we have observed. Finally, an *in vitro* decatenation assay shows that Topo-II is not affected by low doses of CA1 (Supplementary Figure S5A), indicating it is not a target in our experiments. These results support that in HaCaT cells the effects of CA1 on MCM/CMG dynamics are due primarily, if not exclusively, to targeting of the Mcm2-7 ATPases.

### CMGi Disrupt Helicase and Replisome Co-Structural Integrity

**Figure 5:**
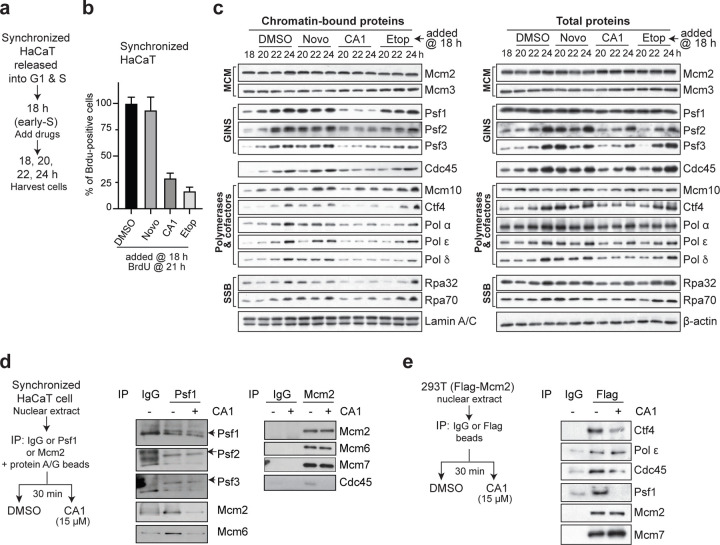
CMGi Disrupt Structural Co-Stability of the hCMG and Replisome in S-phase. (**a**) Experimental design for panels **b** & **c**. (**b**) Synchronized HaCaT cells were released into the cell cycle and allowed to progress into early S-phase (18 hrs). Cells were then treated with compounds and analyzed 3 hrs later for DNA replication using BrdU labeling. Results are averages from three fields, +/− 1s.d. CA1/novobiocin, 15 *µ*M; etoposide, 5 *µ*M; these doses were also used in the following panels. (**c**) Immunoblots of chromatin-bound or total proteins from synchronized HaCaT cells treated with compounds at 18 hr (early S-phase) and assessed at times indicated. (**d**) Experimental design (left) for in vitro assessment of CA1 effects on hCMG complexes. A nuclear extract was prepared from synchronized HaCaT (20 hrs after release; middle S-phase) and subjected to immunoprecipitation with anti-Psf1, anti-Mcm2, or IgG control. Samples were separated in half, then treated with DMSO or CA1 (15 *µ*M) for 30 minutes prior to immunoblotting for (co-)precipitated proteins. (**e**) Experimental design (left) for in vitro assessment of CA1 effects on hCMG and replisome complexes from asynchronous HEK-293T cells stably expressing ectopic Flag-Mcm2. A nuclear extract was prepared and subjected to immunoprecipitation with anti-Flag or IgG as a control. Samples were treated with DMSO or CA1 (15 *µ*M) for 30 minutes and immunoblotted for co-precipitated proteins (or Mcm2).

We determined how CMGi exposure affected the dynamics of hCMG and replisome structure during S-phase. Synchronized HaCaT cells were treated with CA1, novobiocin, or DMSO once cells reached early S-phase (18 hrs) and immunoblots were performed assessing chromatin-bound and total protein components of the replication machinery (Figure 5A). Etoposide has no effect on hCMG activity *in vitro* (Supplementary Figure S5B), and was included to compare how inhibition of Topo-II affected replication dynamics. Treatment with CA1 and etoposide effectively suppressed DNA replication, while novobiocin/DMSO did not (Figure 5B), confirming that hCMG and Topo-II activities are required for ongoing DNA replication. MCM association with chromatin/DNA was not affected by any compounds (Figure 5C, left). However, GINS and Cdc45 chromatin association was notably suppressed by CA1, and not by novobiocin or etoposide. Total protein levels were slightly affected for Psf3 and Cdc45, but not for other subunits (Figure 5C, right). We conclude from these results that DNA topological issues and DNA replication arrest due to Topo-II inhibition do not disrupt GINS/Cdc45 interactions with hCMG helicases. However, CMGi inhibition of ATP binding and/or hydrolysis by the Mcm2-7 ATPases causes GINS and Cdc45 dissociation from hCMG helicases during ongoing DNA replication.

Structural studies have shown that components of the human replisome interact directly with the hCMG helicase, mediated in part through GINS and Cdc45 ^40, 41^. We asked if CMGi-induced loss of Cdc45 and GINS from hCMGs resulted in disruption of replisomes in human cells. Treatment of S-phase cells with CA1, but not other compounds, caused a loss of DNA polymerases-α, -δ, and -ε from chromatin (Figure 5C). Factors such as Ctf4 and Mcm10, which interact with the hCMG and facilitate DNA polymerase-α function on the lagging strand^40, 41^ are also reduced on chromatin by CA1. Consistent with hCMG and replisome disruption, RPA (single-stranded binding protein) is also reduced on chromatin. Intriguingly, exposure to etoposide, which stops DNA replication, does not significantly diminish replisome components or RPA on chromatin, except for a small change to DNA polymerases-α and -δ (Figure 5C). We conclude that, while inhibition of Topo-II (and DNA replication) does not have a significant negative effect on replisome integrity, the structural co-integrity of replisomes and hCMGs is dependent on ATP binding and/or hydrolysis by the Mcm2-7 ATPases during ongoing DNA replication in human cells and is disrupted by CMGi.

We next determined if CMGi treatment of partially purified hCMG helicases and replisomes from human cells displayed similar outcomes. Nuclear extracts were prepared from HaCaT cells enriched in S-phase and subjected to immunoprecipitation using antibodies to Psf1 or Mcm2. Immunoprecipitated complexes were treated directly with CA1 or DMSO, followed by immunoblotting for associated proteins (Figure 5D). Psf1 associates with Psf2, Psf3, Mcm2, and Mcm6, indicating that hCMG helicases were extracted from cells (Figure 5D, middle). We could not examine Cdc45 in this experiment due to signal interference with IgG on immunoblots. Treatment with CA1 did not disrupt Psf1-3 interactions, indicating that the GINS complex itself is not abrogated by CA1. However, Mcm2 and Mcm6 interactions with GINS are abolished by CA1. Mcm2 associates with Mcm6, Mcm7, and Cdc45, and CA1 treatment causes Cdc45 to dissociate from MCMs but does not disrupt MCM complexes (Figure 5D, right). We performed a similar experiment using a different human cell line (HEK-293T) expressing ectopic Flag-Mcm2 (Figure 5E). Flag-Mcm2 interacts with endogenous Mcm7, Psf1, Cdc45, DNA polymerase-ε, and Ctf4 (Figure 5E, right), indicating that Flag-Mcm2 forms complexes with hCMG and replisome components in human cells. CA1 does not disrupt MCM interactions but displaces Psf1, Cdc45, and Ctf4 from Flag-Mcm2. Interestingly, CMGi does not disrupt DNA polymerase-ε binding to Flag-Mcm2, suggesting that differences exist between replisome-hCMG interactions in cells and *in vitro*. A possible explanation is that *in vivo* other factors may contribute to replisome disassembly, such as the ubiquitin ligase CUL2(LRR1)^42^. These experiments again demonstrate that CMGi disrupt the structural co-integrity of hCMG and replisome components. MCM hexamers are not disrupted by CMGi, consistent with yeast studies using MCM ATPase mutants 29, 30. However, interactions of GINS, Cdc45, DNA polymerases, and co-factors with hCMG helicases depend on functional MCM ATPase domains during S-phase that are inhibited by CMGi exposure.

### CMGi Cause DNA Damage, Checkpoint Activation, and Apoptosis

**Figure 6:**
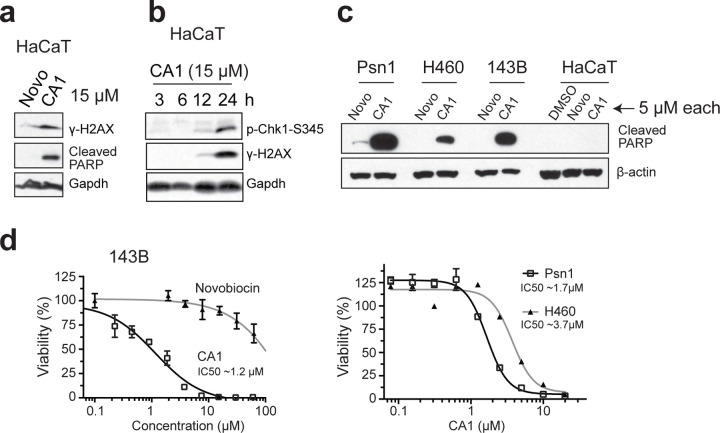
CMGi Induce DNA Damage/Apoptosis, and Tumor Cells Are Selectively Sensitive. (**a**) Immunoblot of total proteins from HaCaT cells treated with 15 *µ*M each of novobiocin or CA1 for 24 hrs. (**b**) Immunoblot of total proteins from HaCaT cells treated with CA1 (15 *µ*M) for the number of hours indicated. (**c**) PARP cleavage was assessed by immunoblotting total protein samples from pancreatic ductal adenocarcinoma (Psn1), non-small cell lung cancer (H460), and osteosarcoma (143B) tumor lines to HaCaT samples after 5 *µ*M CA1 or novobiocin (or DMSO carrier) treatment for 24 hrs. (**d**) Viability analyses were performed on 143B, Psn1, and H460 tumor lines to determine sensitivity to CA1 or novobiocin (only on 143B). IC50 determinations for CA1 are indicated.

We reasoned that loss of replisome and hCMG integrity would stall replication forks and create DNA damage. To test this, we treated HaCaT cells with 15 *µ*M novobiocin or CA1 and asked if surrogate measures of DNA damage and apoptosis were present. The data in Figure 6A shows that gamma-H2AX levels increase after 24 hr exposure to CA1, but not novobiocin. CA1 treatment also causes increased cleavage of PARP, indicative of apoptosis (Figure 6A), and causes an increase in phosphorylated Chk1 (Figure 6B). We conclude that CMGi-induced loss of hCMG and replisome structural integrity is associated with increased DNA damage, checkpoint signals, and apoptosis.

### Tumor Cells Are Selectively Sensitive to CMGi

CMG helicases and reserve MCMs may be weakened in tumor cells due to oncogene-driven changes ^1^, which would render tumor cells more sensitive to CMGi relative to non-tumor cells. To test this, we compared three tumor lines to HaCaT cells for their sensitivity to a lower dose of CA1 or novobiocin (24 hr exposure, 5 *µ*M) by assessing PARP cleavage. All three tumor lines displayed significant PARP cleavage only in the presence of CA1, while HaCaT showed no effect at this lower dose (Figure 6C). Viability analyses determined that tumor lines were ~4–10 times more sensitive to CA1 exposure relative to HaCaT, with IC_50_ estimates of 1–4 *µ*M (Figure 6D). These results demonstrate that tumor cells are selectively sensitive to CA1/CMGi exposure, and that the CMG helicase (or MCM complexes) is a novel target for anti-cancer intervention.

## DISCUSSION

### Mode of hCMG Binding and Inhibition by CMGi

We have identified the first small chemical compounds capable of inhibiting ATPase and helicase functions of the human replicative CMG helicase. Clorobiocin and CA1 are effective and selective CMGi at low concentrations, competing with ATP for binding and hydrolysis at one or more ATPase sites within the MCM ring. SAR suggests selectivity of CA1 derives from the composition of the noviose sugar groups. Consistent with the mechanism of binding and ATP-competitive inhibition by these compounds toward their bacterial target, gyrase^18, 27^, CA1 and clorobiocin bind channels within MCM subunits leading to particular ATPase clefts. The sugar groups occupy space where adenosine and ribose of ATP normally interact, thereby producing a direct competition for ATP binding. Modeling also suggests that these inhibitors compete with ATP through a steric block to ATP/ADP ingress or egress through channels at the ATPase domains. Thus, these CMGi act like a ‘cork in a wine bottle,’ but one that also displaces the ‘wine’ (i.e., ATP).

The existing cryo-EM structural data for the hCMG^31^ allows only three ATPase sites to be docked by CMGi (Mcm3-7, Mcm4-6, and Mcm5-3). Since the other three MCM ATPase channels/clefts cannot be docked with CMGi due to steric hindrance, two somewhat mutually exclusive interpretations emerge. In one case, CMGi may never be capable of binding these clefts if the channels remain in a non-permissible conformation. A corollary of this would require that such conformations not restrict ATP/ADP movement through the narrow channels. Alternatively, there may be certain enzyme states not observed in this cryo-EM structure during which channels become wider and accessible to CMGi entry. Such a molecular ‘breathing’ model would suggest that ATP/ADP movement at each ATPase domain might be controlled by altering the width of these channels, and thus ATP hydrolysis around the MCM ring might be regulated through allosteric ATP accessibility.

At present we cannot determine whether CMGi have a preference for binding certain ATPase clefts, perhaps rendered accessible during MCM loading, hCMG assembly, or activation of the helicase. Studies of the *Drosophila* CMG demonstrate that mutation of individual ATPase clefts influences ATPase activity of other clefts ^4^. Thus, MCM ATPase domains use allosteric means to affect ATPase function of other sites. CMGi binding and inhibition at specific MCM ATPase domains may also allosterically alter and inhibit ATPase activity of other sites. In support of this, CMGi exposure induces allosteric changes in the MCM ring that cause the cofactors GINS and Cdc45 to dissociate. GINS and Cdc45 allosterically stimulate ATPase functions of the MCM ring^4^. However, the converse is also true, that inhibition of the ATPase domain(s) of the MCM ring by CMGi causes a ‘reverse’ allosteric dissociation of the same cofactors. There is thus a structural and functional integrity of the hCMG holoenzyme that is interdependent on the association of GINS/Cdc45 with MCM rings.

### Mechanisms of CMGi on ATP-dependent MCM/CMG Functions in Human Cells

Mutation of the majority of *S. cerevisiae* MCM (ScMCM) ATPase clefts results in loss of viability, derived in part from loss of ScMCM loading onto DNA^28, 29, 30, 43^. In some cases, mutation of specific ATPase sites allows for partial loading of ScMCM complexes that are then deficient for GINS recruitment or unwinding steps of DNA replication^30^. ATP binding is necessary to maintain stable GINS and Cdc45 association with ScCMG complexes after ScMCM loading^30, 34^, and double-CMG formation involves changes to ATP/ADP interactions at specific ScMCM ATPase sites^44^.

Consistent with ScMCM studies, loading of human MCM (hMCM) onto chromatin/DNA in human cells is dependent on hMCM ATPase domain functionality and inhibited by CMGi. We cannot assign a particular ATPase domain of the hMCM ring to this dependency and CMGi sensitivity, nor can we specify that this is due to ATP binding, hydrolysis, or both. CMGi dock in three sites of the hCMG that correspond to sites in the ScMCM that require ATP binding and/or hydrolysis for efficient DNA loading^29, 30^. Our human cell results contrast with a *Xenopus* study in which mutagenesis of Mcm6 and Mcm7 ATPase domains did not affect MCM hexamer loading.^45^ As an explanation, CMGi may target different ATPase clefts of the hMCM that are required for loading. After hMCM loading in human cells, and consistent with yeast studies, GINS is sensitive to abrogation of hMCM ATP binding and/or hydrolysis by CMGi during hCMG assembly.

During DNA replication, inhibition of hCMG ATPase domains by CMGi causes disruption of replisomes. In contrast, exposure to etoposide only stops DNA replication, without disruption of helicase and replisome integrity. The unique disruption to replisome structure by CMGi is likely attributable to effects on hCMG integrity, as CMGi causes dissociation of the helicase cofactors, GINS and Cdc45. Structural determinations by cryo-EM of human replisomes indicates that Ctf4, which interacts with DNA polymerase-α^41^, and DNA polymerase-ε make contacts with the hCMG through interactions with GINS and Cdc45.^40^ Our observations that loss of GINS/Cdc45 from hCMGs after CMGi treatment also disrupts replisome integrity is consistent with these structural findings. CMGi are thus mechanistically distinct from other DNA replication-inhibiting drugs in their mode of action, causing hCMG/replisome disruption.

Replisome structural integrity is dependent on ATP binding and/or hydrolysis by the hCMG ATPase domains, as demonstrated by replisome sensitivity to CMGi. The hCMG is thus a molecular platform for maintaining replisome integrity during DNA replication, and dismantling of replisomes can be achieved by abrogating the ATPase domains of the hCMG. It is interesting to speculate that cells might possess a soluble factor that is capable of targeting the hCMG ATPases (directly or via post-translational modifications), leading to replisome disruption during intra-S checkpoints or DNA repair at stalled forks, or when replisomes from approaching forks in adjacent replicons meet. CMGi will be a valuable chemical probe to further interrogate these hCMG and replisome functions in mammalian cells.

### CMGi Define a New Class of Anti-Cancer Compounds

Common targets in anti-cancer regimens often include the DNA replication and repair machinery. Clearly, the CMG helicase represents another such target, and the CMGi discovered here will inform the development of additional derivatives for use in the anti-cancer arsenal. Aminocoumarins were originally marketed or investigated in clinical trials as anti-bacterial agents, but their use was supplanted by quinolones such as ciprofloxacin ^18^. However, aminocoumarins such as these CMGi may offer clinical advantages with novel modes of action in suppressing tumor growth due to targeted inhibition of the CMG and replisomes. More importantly, oncogenic changes mismanage the regulation of reserve MCMs/CMGs, predicting deficiencies in CMG functionality and a tumor-specific vulnerability in the CMG relative to non-tumor cells ^1, 46^. In support of this, our results show that tumor cells are indeed selectively sensitive to CMGi, and, importantly, that a therapeutic window exists for targeting the CMG helicase with CMGi.

Consistent with our findings, the National Cancer Institute has publicly available data showing that many solid tumor cell lines (NCI-60 set) are highly sensitive to growth suppression by the CMGi identified here (CA1, NSC107412; clorobiocin, NSC227186), but are insensitive to novobiocin (NSC2382; https://dtp.cancer.gov/dtpstandard/dwindex/index.jsp). Until now, a cellular target consistent with these differences was not clear. It had been suggested that HSP90 might be a target, but the high doses required to inhibit HSP90 (700–1000 *µ*M) and its sensitivity to CA1 and novobiocin suggest it is not the target^47, 48^. The direct relationship between the low doses that inhibit the CMG in biochemical assays, and similar low doses that inhibit the CMG *in vivo* to arrest cells, strongly suggests that the CMG is a major target of these CMGi. Interestingly, novobiocin can inhibit a different anti-cancer target, DNA Polymerase-theta, which also contains a helicase domain and is involved in DNA repair ^49^. The inability of novobiocin to inhibit the CMG demonstrates that these aminocoumarins display target specificity and distinct modes of action. Given the many roles for MCM/CMG complexes in cell cycle regulation, DNA replication, and DNA repair, future CMGi with drug-like qualities and distinct mechanisms of action will provide a novel means for cancer intervention.

## Online Methods:

### Cell Lines and Inhibitors

Human keratinocytes (HaCaT; RRID:CVCL_0038) and HEK-293T (RRID:CVCL_0063) cells were maintained in Dulbecco’s Modified Eagle’s Medium (DMEM) supplemented with 10% fetal bovine serum (FBS; Peak Serum, PS-FB-2). HaCaT cells were synchronized in G_0_ using serum starvation for 48 hr and released into the cell cycle by addition of DMEM with 10% FBS ^50^. Novobiocin (cat# 46531) and Courmermycin-A1 (cat# C9270) were obtained from Sigma. Etoposide (cat# S1225) and AT7519 (cat# S1524) were obtained from SelleckChem. All stock solutions of inhibitors were stored at −20^o^C as 10 mM suspensions in DMSO.

### Cell Viability Assays

Cell viability determinations were performed using CellTiter-Glo Assays (Promega; cat# G7572). Cells were seeded in 96-well plates at a density of ~3 × 10^3^ cells/well and treated with drugs for 72 hr, after which the cells were processed for viability using CellTiter-Glo reagent according to the instructions of the manufacturer. Each drug concentration test was performed using four replicates, and results averaged and plotted on graphs, +/− 1 s.d.

### Immunoblotting and Antibodies

Immunoblotting was performed using standard enhanced chemiluminescent (ECL) and polyacrylamide gel techniques. Lysates from equal cell numbers were separated into Triton X-100-soluble or -resistant (chromatin-bound) protein fractions as described ^51, 52^, and compared to whole-cell protein lysates. All cell lysates were supplemented with protease inhibitors (1 mM PMSF, 1 mM benzamidine, 0.15 *µ*M Aprotinin, 4 *µ*M Leupeptin, 1 *µ*M Antipain). Immunoblots were assessed with the following antibodies (all used at 1:500–1:1000 dilutions): from Santa Cruz: anti-Mcm5 (sc-165994, RRID:AB_2142526), anti-Mcm6 (sc-55577, RRID:AB_831540), anti-Mcm7 (sc-9966, RRID:AB_627235), anti-Orc2 (sc-32734, RRID:AB_2157726), anti-Cdt1 (sc-28262, RRID:AB_2076885), anti-Cdc6 (sc-9964, RRID:AB_627236), anti-phospho-Ser54-Cdc6 (sc-12920-R, RRID:AB_668066), anti-DNA polymerase ε (sc-12728, RRID:AB_675496), anti-DNA polymerase δ (sc-17776, RRID:AB_675487), anti-γ-H2AX (sc-517348, RRID:AB_2783871), anti-Sld5 (sc-398784, RRID:AB_2940776); from Abcam: anti-Psf1 (ab183524, RRID:AB_2922402), and anti-Psf3 (ab254855, RRID:AB_2940777), anti-phospho-Ser53-Mcm2 (ab109133, RRID:AB_10863901), anti-DNA polymerase α (ab31777, RRID:AB_731976); from Proteintech: anti-Psf2 (16247-1-AP, RRID:AB_2111895), anti-GAPDH (60004-1-lg, RRID:AB_2107436); from Cell Signaling: anti-RPA32 (2208s, RRID:AB_2238543), anti-RPA70 (2267s, RRID:AB_2180506), anti-Mcm3 (4003s, RRID:AB_2142261), anti-Lamin A/C (2032S, RRID:AB_2136278), anti-WRN (4666, RRID:AB_10692114), anti-BLM (2742, RRID:AB_2064649), anti-phospho-Ser139-Mcm2 (12958, RRID:AB_2798069), anti-phospho-Ser345-Chk1 (2348, RRID:AB_331212), anti-cleaved Parp (5625, RRID:AB_10699459), anti-phospho-Thr320-PP1-α (2581, RRID:AB_330823), anti-Rb (9313, RRID:AB_1904119), anti-phospho-Ser807/811-Rb (9308, RRID:AB_331472); from Sigma-Aldrich: anti-Flag (F3165, RRID:AB_259529), anti-beta-actin (A5441, RRID:AB_476744), rabbit IgG (I5006, RRID:AB_1163659), anti-Flag agarose (A2220, RRID:AB_10063035), anti-Mouse IgG agarose (A6531, RRID:AB_258295); from Biolegend: anti-And-1 (Ctf4) (630301, RRID:AB_2215084); from Bethyl Laboratory: anti-Mcm10 (A300-131A, RRID:AB_2142119); from Invitrogen: anti-PP1-α (MA5-15589, RRID:AB_10980092); from BD Pharmingen: anti-Mcm4 (559544, RRID:AB_397267). Chicken polyclonal anti-Cdc45, rabbit polyclonal anti-Cdc6, and rabbit polyclonal anti-Mcm2 (used at 1:2000 dilutions) were generated by our group and validated as described ^8^.

### BrdU Labeling and Immunofluorescence Techniques

Verification of synchronization and determination of drug effects was performed by measuring the incorporation of bromo-deoxyuridine (BrdU) into replicating foci within nuclei. At times indicated, cells were pulse-labeled with 15 *µ*M BrdU for 30 min, fixed with 4% paraformaldehyde, and analyzed by standard immunofluorescent techniques^36, 50^ with an anti-BrdU monoclonal antibody (Roche, clone no. BMC9318, RRID:AB_2313622). The average counts of three fields of 100 or more cells were used to determine the percentages of BrdU-labeled nuclei, +/− 1 s.d.

### Topoisomerase II Decatenation Assay

The Topoisomerase II drug screening kit (kDNA-based; TopoGEN, TG1009-1A) was used to assess *in vitro* Topo II enzyme activity according to the manufacture protocol. Assays were performed in 20 μL reactions with 4 *µ*L of 5X Assay Buffer (0.25 M Tris-HCl (pH 8), 10 mM ATP, 0.75 M NaCl, 50 mM MgCl_2_, 2.5 mM Dithiothreitol, 150 μg/ml BSA), 1 μL kDNA (0.2 μg), 1 μL DMSO solvent or compounds at indicated concentrations, 1 μL Topoisomerase II (2 Units), 13 μL water. Assays were incubated at 37°C for 30 min and stopped by addition of 2 μL 10% SDS and Proteinase K (50 μg/ml). After incubating at 37°C for 15 min, samples were mixed with 1/10 volume of loading buffer and resolved on a 1% agarose gel.

### Bloom (BLM) and Werner (WRN) Helicase siRNA-Mediated Knockdowns

HaCaT cells were transiently transfected using the Lipofectamine RNAiMAX reagent (cat# 13778; Invitrogen) with 20 nM SMARTpools of Human BLM siRNA (L-007287-00-0005), Human WRN siRNA (L-010378-00-0005), or non-targeting siRNA (NT, D-001810-02-05) pool#2 (all from Dharmacon). Transfected cells were collected 72 hrs after transfection and subjected to immunoblotting analyses.

### Co-Immunoprecipitation Assays

Nuclear extracts were prepared from synchronized HaCaT cells after releasing into S-phase (at 20 hrs post release) or HEK-293T cells expressing stable Flag-Mcm2 protein (human). Cells from one 10 cm dish were resuspended in 300 μL of Buffer A (10 mM Hepes-KOH pH 7.5, 10 mM KCl, 1.5 mM MgCl_2_, 0.34 M sucrose, 10% glycerol, protease inhibitors, and 0.1% Triton-X-100) and incubated on ice for 5 min. The nuclear pellet was obtained by centrifugation at 4,000 rpm at 4°C for 5 min and washed with the Buffer A without Triton X-100. The nuclear extract was obtained by resuspending the nuclear pellet in 300 μl of Buffer A containing 420 mM potassium acetate and 0.01% Triton X-100, and incubating at 4°C for 1 hr. The final concentration of potassium acetate in nuclear extracts was adjusted to 200 mM for the co-immunoprecipitation assays. For the co-immunoprecipitation assays, 10 μg of indicated antibody [anti-Mcm2, anti-Psf1, or rabbit IgG (Sigma)], or 30 μL of mouse IgG or anti-Flag agarose beads (Sigma), was incubated with 300 μL of nuclear extract at 4°C for 4 hrs. Protein A/G agarose beads (30 μL, Santa Cruz, sc-2003) were added and incubated for 1 hr with antibodies. Agarose beads were washed once in Buffer A containing 200 mM potassium acetate and 0.01% Triton X-100 and incubated with 15 *µ*M CA1 or DMSO (same concentration as in CA1 sample) for 30 min. Beads were washed in the same buffer and analyzed by immunoblotting with indicated antibodies.

### Purification of the Human CMG Helicase

The human CMG helicase was purified following the established and validated protocol of Hurwitz and colleagues^19, 20^, with minor modifications. A detailed description of the approach is shown here, to allow for comparisons to the methods described by the Hurwitz group.

High Five insect cells (2–3 million cells/ml, 1.5 L), were in cultured in a shaking incubator at 27°C in suspension with ESF921 serum free medium (Expression Systems, CA; cat. #96-001-01), then co-infected for 60 hrs with 11 baculoviruses expressing human 6His2Flag-Cdc45, the human MCM hexamer (Mcm2, Mcm3, Mcm4, Mcm5, Mcm6, Mcm7), and human GINS (GST-Sld5, Psf3, Psf2, and Psf1). Each virus was infected at an MOI of ~10 from individual virus stocks. Infected cells were harvested by centrifugation at 650×g for 5 min at 4°C, washed with cold PBS, frozen on dry ice, and stored at −80°C until use. The cell pellet (~20 mL) was thawed on ice, resuspended in 45 ml Hypotonic Buffer [20 mM Hepes-NaOH (pH 7.5), 5 mM KCl, 1.5 mM MgCl_2_] with protease inhibitors (1 mM PMSF, 1 mM Benzamidine, 0.15 *µ*M Aprotinin, 4 *µ*M Leupeptin, and 1 *µ*M Antipain), and kept on ice for 10 min before lysing by Dounce homogenization (tight fitting, 60 strokes). The cell extract was adjusted to 0.42 M potassium acetate and centrifuged at 43,000×g for 1 hr at 4°C. The cleared lysate was mixed with 0.75 ml glutathione beads (cat. #17-0756-05; GE Healthcare) pre-equilibrated with FEQ buffer [20 mM Hepes-NaOH (pH 7.5), 0.42 M potassium acetate, 5 mM KCl, 1.5 mM MgCl_2_] and incubated by rotation at 4°C overnight. Following centrifugation at 290xg at 4°C, the bound glutathione beads were washed four times (15 min each wash) with 40 ml of FW buffer [20 mM Hepes-NaOH (pH 7.5), 0.42 M potassium acetate, 1 mM DTT, 1 mM EDTA, 0.01% NP40, 10% glycerol (vol/vol) with protease inhibitors as above] containing protease inhibitors (as above). Bound proteins were eluted at 4°C three times (1 hr each elution) with 3.5 ml Q buffer [20 mM Hepes-NaOH (pH 7.5), 1 mM DTT, 1 mM EDTA, 0.01% NP40, 10% glycerol (vol/vol), and protease inhibitors as above] containing 0.15 M potassium acetate and 20 mM reduced glutathione. The eluted fractions were combined and applied to a HiTrap Q-Sepharose FF column (HiTrap Q FF 1 mL, cat. #17505301, Cytiva), pre-equilibrated 3X with 5 ml of Q buffer containing 0.15 M, 0.75 M, 0.15M potassium acetate, in sequence. The Q-Sepharose FF column was washed with 10 ml of FW buffer containing protease inhibitors (as above). The proteins were eluted with 10 ml of Q buffer containing 0.75 M potassium acetate. The eluted fraction was mixed with 0.15 ml of anti-Flag M2 affinity gel/beads and incubated while rotating at 4°C overnight. After centrifugation at 290×g for 5 min at 4°C, the beads were washed three times with 10 ml PreScission buffer [50 mM Tris-HCl (pH 7.5), 0.15 M NaCl, 1 mM DTT, 1 mM EDTA] for 15 min at 4°C, and eluted at 4°C three times (1 hr each elution) with 0.2 ml PreScission enzyme buffer containing 0.2 mg/ml 3X-Flag peptides. The combined eluates were incubated with 20 Units of PreScission Protease (GE Healthcare) and 0.1 mL glutathione beads for 4 hr at 4°C. The supernatant was layered onto a 15–40% glycerol gradient [25 mM Tris-HCl (pH 7.5), 50 mM NaCl, 1mM DTT, 1mM EDTA, 0.01% NP40, protease inhibitors as above] in a 5 mL ultra-centrifugation tube and centrifuged at 260,000×g for 14 hr at 4°C. Glycerol fractions (0.15 mL each fraction) were collected from bottom of tube and stored at −80°C until use. Typically, fractions 6–9 contained complete hCMG (~750–800 kDa) and co-migrated with thyroglobulin.

Estimation of hCMG protein amount isolated was determined by comparing to BSA standards. We nominally achieved purification of 5–6 ng/*µ*L hCMG enzyme from three glycerol fractions, or ~7.5 fmol hCMG/*µ*L. Across multiple preps this varied from 5–15 fmol hCMG/*µ*L, consistent with that reported by Hurwitz and colleagues ^19, 20^. The specific ATPase activity of our isolated hCMG enzyme was consistent with the hCMG ATPase activity obtained by Hurwitz and colleagues ^19, 20^. This prior hCMG analysis determined that the human helicase hydrolyzes ATP to ADP at a rate of ~80 mol-ADP per minute per mol-hCMG in the presence of 500 *µ*M ATP. Using the ADP-sensing fluorescent-polarization assay (described below), 15 fmol (in 2 *µ*L) of hCMG elicits an ~52% mP change relative to the assay window (see Results), equating to production of ~7–8 *µ*M ADP in 1 hr (80 × 15 fmol x 60 min = 72 pmol ADP in a 10 *µ*L reaction, or 7.2 *µ*M ADP). This activity represents an ~1.5% ADP conversion rate by the hCMG and falls within the reliability range of Z’ = 0.6–0.8 for screening in the primary ATPase assay.

### Helicase Assays

Helicase fork-unwinding assays were performed at 37°C for 1 hr in 20 *µ*L reaction volumes using 2–4 *µ*l of hCMG (~15–30 fmol), in a buffer consisting of 25 mM Hepes-NaOH (pH 7.5), 5 mM NaCl, 0.5 mM ATP, 10 mM magnesium acetate, 1 mM DTT, 0.1 mg/ml bovine serum albumin (BSA), and ~10 fmol of radiolabeled DNA forks ^20^. Reactions were terminated with 4 *µ*l of 6X stop solution [50 mM EDTA (pH 8.0), 40% (vol/vol) glycerol, 2% (wt/vol) SDS, and 0.3% bromophenol blue], loaded onto 10% (wt/vol) polyacrylamide gels, resolved at 150 volts in 1X-TBE buffer (89 mM Tris base, 89 mM boric acid, and 2 mM EDTA), dried on filter paper, and analyzed by autoradiography, densitometry, and phosphorimager assessment.

The DNA substrates were formed as described by Hurwitz and colleagues ^20^ by annealing two oligonucleotides: 10 pmol of M13–39–5′dT40

(5′-(T)_40_GATTAAGTTGGGTAACGCCAGGGTTTTCCCAGTCACGAC-3′)

and 10 pmol of anti-M13-39–3′dT40

(5′-GTCGTGACTGGGAAAACCCTGGCGTTACCCAACTTAATC(T)_40_-3′)

in the presence of 0.1 M NaCl by heating for 5 min at 95°C, followed by slow cooling to room temperature. The oligonucleotide M13-39–5′dT40 was 5’ end-labelled with T4 Polynucleotide Kinase (cat# M0201S; New England Biolabs) and [γ−^32^P]-ATP before annealing. The annealed DNA substrates were resolved in 10% polyacrylamide gels in 1X-TBE buffer at 150 volts for 30 min. Bands containing double-stranded DNA forks were cut from the gel and forks eluted after crushing the gel with 200 *µ*L Tris-EDTA buffer containing 0.15 M NaCl for 3 hr at 37°C. The eluted fraction was collected by centrifugation at 13,000 rpm at 4 °C for 10 min and stored at 4°C until use.

### Fluorescent-Polarization Measurements with ADP^*2*^ Transcreener Assay

The fluorescent-polarization (FP) ADP-sensing assays were performed using the Transcreener^®^ ADP^2^ FP assay kit (cat# 3010-1K, Bellbrook Labs). For hCMG inhibitor screening, assays were performed at 37°C for 1 hr in 10 *µ*l reactions in a 384-well plate (cat. #4514, Corning) using 2 *µ*L of hCMG (~15 fmol), 25 mM Hepes-NaOH (pH 7.5), 10 mM NaCl, 0.5 mM ATP, 10 mM magnesium acetate, 1 mM DTT, 0.1 mg/ml BSA, and DNA fork substrates. But assays without DNA substrates can also be performed, since the hCMG does not require DNA to be present to hydrolyze ATP ^20^. Selected inhibitors or samples from a chemical library were added into the reactions when conducting the screening or testing inhibitor effectiveness. The NCI Diversity Set VI chemical library was obtained from the National Cancer Institute (Bethesda, MD).

The window of sensitivity for the ADP-sensing assays is determined by setting up two 10 *µ*L control samples without any added hCMG helicase: a Low-FP mixture (4 nM ADP Alexa Fluor-633 Tracer alone) and a High-FP mixture (4 nM ADP Alexa Fluor-633 Tracer plus patented anti-ADP^2^ Antibody). The amount of ADP^2^ Antibody used had to be adjusted to account for the use of 500 *µ*M ATP in our hCMG assays, versus 10 *µ*M ATP in enzyme reactions typically assessed with standard kits prepared by BellBrook Labs. The ADP^2^ Antibody has a significantly higher affinity for ADP compared to ATP, but since it can bind to ATP to some extent, this must be offset by including more ADP^2^ Antibody in our high-ATP assays. This was done according to the manufacturer by performing a titration with increasing ADP^2^ Antibody, fixed 4 nM ADP Alexa Fluor-633 Tracer, and 500 *µ*M ATP to determine the ~EC_80–85_ for millipol (mP) changes, which determined the optimal ADP^2^ Antibody concentration to use as 0.64 mg/ml (Supplementary Figure S2A). Samples are read with a Perkin Elmer Envision II plate reader with optimized Cy5 (far-red) FP-compatible mirror and cubes (cat# 2100-8390, Perkin Elmer). The Low-FP sample is the least polarized and gives a low mP reading, while the High-FP sample is the most polarized and gives the highest mP reading. The difference between the Low-FP and High-FP values defines the FP assay window, which is normally in a range of 150–200 mP under ideal conditions for screening purposes. The hCMG helicase hydrolyzes ATP to ADP and decreases the mP reading within this window, with an ideal change (∆mP) in the FP window of at least 50% to be in a readable range for inhibitor screening (as per manufacturer). Potential hCMG chemical inhibitors reverse this effect and cause the mP readings to increase.

We performed a titration with increasing and decreasing concentrations of ADP and ATP, respectively, to assess the sensitivity of the FP assay in detecting ADP production (Supplementary Figure S2B). Consistent with the stated manufacturer predictions, the assay can reliably detect 1–3% changes in ADP production (*i.e.*, 5–15 *µ*M ADP production) in starting concentrations of ATP of 500 *µ*M, with a Z’ efficiency between 0.6–0.8 (determined following manufacturer protocols). We also note that ATP-gamma-S cannot be used as a positive control for inhibition of the CMG in this ADP-sensing assay, as it competes with the anti-ADP^2^ Antibody and alters the detection window by itself (Supplementary Figure S2C).

Once the hCMG enzyme reactions are complete, 10 *µ*L of 1X Stop Solution and Detection Buffer (buffer from BellBrook Labs; containing 4 nM ADP Alexa Fluor-633 Tracer plus ADP^2^ Antibody) are added to each reaction, and to the High-FP control. The Low-FP control receives 10 *µ*L of 1X Stop Solution and Detection Buffer containing only 4 nM ADP Alexa Fluor-633 Tracer (no Antibody). Samples are incubated at 25°C for 1 hr, then read in the plate reader. Potential positive inhibitors (hits) of the hCMG are verified to be incapable of altering the mP window on their own, by artificially raising the mP readings to appear more polarized as occurs when the hCMG is truly inhibited by a compound. For this, potential hCMG inhibitors are added to a 10 *µ*L mixture containing 25 mM Hepes-NaOH (pH 7.5), 10 mM NaCl, 0.5 mM ATP, 10 mM magnesium acetate, 1 mM DTT, 0.1 mg/ml BSA, and no hCMG, and mixed with 10 *µ*L of Stop Solution and Detection Buffer (4 nM ADP Alexa Fluor^®^ 633 Tracer without ADP^2^ Antibody). Plate readings from these tests of potential positive hits should be similar to that seen with the Low-FP control, or such hits may instead be false-positives.

### *In silico* Docking Parameters

The structure of the human CMG helicase determined at 3.3 Angstroms by electron cryomicroscopy (cryo-EM) with bound ADP, ATP-gamma-S, or no nucleotide within individual ATP clefts, and with bound DNA (PDB accession code 6XTX) was used for *in silico* docking with Autodock Vina Version 1.2.3 (RRID:SCR_011958) ^31^. Estimated interaction/binding energies were calculated by Autodock, and the outcomes converted to Kd estimates using the formula ∆G = RT(ln)(Kd), with T set at 37^o^C/310^o^K. The conversion of protein and small chemical molecules into pdbqt format and grid box generation were achieved by using Autodock Tools Version 1.5.6. Each pair of MCMs forming an ATP cleft was extracted and the grid box derived with the active site in the center of the cleft. The docking configurations of clorobiocin were viewed and analyzed using Pymol Version 2.4.1 (RRID:SCR_000305).

### Quantification and Statistical Analysis

All the statistical details of experiments can be found in the figure legends or Methods descriptions. Statistical analyses were performed with Prism (GraphPad). Error bars represent mean ± SD. Statistical comparisons were analyzed with unpaired two-tailed Student’s t-test. Data were considered statistically different at P < 0.05.

## Supplementary Material

Supplement 1
