# Supplementary material for: Identification of Selective ATP-Competitive CMG Helicase Inhibitors for Cancer Intervention that Disrupt CMG-Replisome Function
Source: Res Sq. 2023 Aug 11:rs.3.rs-3182731. Preprint. [Version 1] doi: 10.21203/rs.3.rs-3182731/v1 (PMC10441460; doi:10.21203/rs.3.rs-3182731/v1)
Supplement: Supplement 1 [file NIHPPrs3182731v1-supplement-1.pdf]

Supplementary Files

This is a list of supplementary files associated with this preprint. Click to download.

- SupplementaryFigures.pdf
